# Piperine protects against cerebral ischemic injury by regulating the Caspase-1-mediated pyroptosis pathway

**DOI:** 10.3389/fphar.2025.1601873

**Published:** 2025-07-25

**Authors:** Jiayuan Lu, Xinwen Dai, Siyu Xi, Bo Wang, Peng Zhang, Xueyan Fu, Juan Liu, Yiwei Zhang

**Affiliations:** ^1^ School of Basic Medical Sciences, Ningxia Medical University, Yinchuan, China; ^2^ Key Laboratory of Ningxia Ethnomedicine Modernization, Minority of Education, Ningxia Medical University, Yinchuan, China; ^3^ School of Clinical Medicine, Ningxia Medical University, Yinchuan, China; ^4^ School of Pharmacy, Ningxia Medical University, Yinchuan, China; ^5^ General Hospital of Ningxia Medical University, Yinchuan, Ningxia, China

**Keywords:** natural product, cerebral ischemic injury, neuroprotective, pyroptosis, Caspase-1

## Abstract

**Background:**

Ischemic stroke (IS) is a prevalent form of stroke and marked by high rates of morbidity, disability, and mortality. IS greatly threatens the physical health of people around the world. Oxidative stress triggered by IS can lead to inflammatory responses. Piperine (Pip) is a bioactive dietary phytochemical known for its pharmacological properties, including anti-inflammatory, anti-tumor, and antioxidant effects. Pip has attracted considerable interest among researchers. This study aims to investigate whether Pip attenuates cerebral ischemic injury by regulating the Caspase-1-mediated pyroptosis pathway.

**Methods:**

*In vivo* and *in vitro* experimental models were employed. For the *in vivo* simulation of cerebral ischemia, the rat permanent middle cerebral artery occlusion (pMCAO) model was utilized. For the *in vitro* simulation, the BV-2 cells were subjected to oxygen–glucose deprivation (OGD). The recovery of neurological function in rats was assessed through multiple behavioral tests, including the Zea-Longa score, balance beam test, traverse beam test, forelimb grip pull test, postural reflex test, sensory test, and tail lifting test. Pathological changes in cerebral ischemic injury were observed using TTC staining, HE staining, and transmission electron microscopy. In *in vivo* and *in vitro* experiments, the potential protective mechanism of Pip in alleviating cerebral ischemic injury by regulating the Caspase-1-mediated pyroptosis pathway was investigated using Western blot and reverse transcription-polymerase chain reaction assays.

**Results:**

In the *in vivo* experiments, compared with the Sham group, the Model group exhibited significant neurological damage, increased infarct volume, brain tissue edema, and elevated protein and mRNA expression levels of pyroptosis-associated factors. By contrast, the Pip group demonstrated notable improvements in behavioral function, brain tissue morphology, and the expression levels of pyroptosis-related factors compared with the Model group. In the *in vitro* experiments, the protein and mRNA expression of pyroptosis-associated factors in the OGD group were significantly upregulated compared with that in the Con group. However, the expression of these factors in the OGD+Pip group was markedly reduced compared with that in the OGD group. Furthermore, when cells were treated with the Caspase-1 inhibitor Ac-YVAD-cmk, the results revealed a significant decrease in the protein expression of Caspase-1 and its downstream factors, GSDMD-N and IL-1β, compared with that in the OGD group. Notably, the protein expression of GSDMD-N and IL-1β in the Pip+Ac-YVAD-cmk group was significantly higher than in the Pip group, which suggests that the inhibition of Caspase-1 attenuated the suppressive effect of Pip on GSDMD-N and IL-1β expression.

**Conclusion:**

Pip exerts neuroprotective effects by modulating the Caspase-1-mediated pyroptosis pathway, which inhibits neuronal damage in the pMCAO model. These findings highlight the therapeutic potential of Pip in mitigating cerebral ischemic injury.

## Graphical Abstract

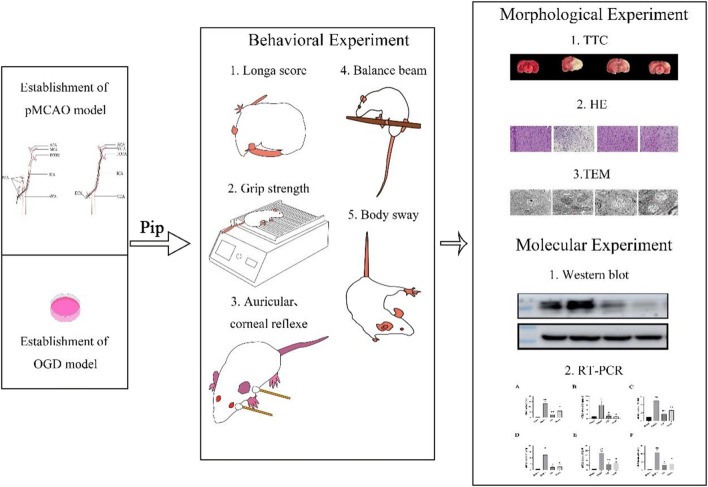

## Highlights

• *In vivo* studies demonstrated that Pip exerts neuroprotective effects and mitigates ischemic brain tissue damage in the affected hemisphere, as evidenced by: Behavioral assessments, Histopathological staining, and TEM.
• *In vitro* experiments revealed that Pip significantly downregulated the expression of pyroptosis-related factors, such as Caspase-1 and GSDMD-N, based on molecular biology analyses.

## 1 Introduction

Stroke is commonly known as cerebral apoplexy or cerebrovascular disease; it is a prevalent and devastating disease that affects the global population and ranks as the second leading cause of death worldwide (Johnson et al., 2019). Stroke is primarily categorized into two main types: ischemic stroke (IS) and hemorrhagic stroke, with IS accounting for approximately 80% of all stroke cases globally (Sun et al., 2019). IS is characterized by high rates of morbidity, disability, and mortality; it imposes a significant economic burden on patients and healthcare systems (Wen et al., 2020).

IS is primarily caused by an acute cerebrovascular injury resulting from the blockage of blood vessels (Peisker et al., 2016) which leads to the formation of an ischemic region in the brain where glucose and oxygen supply is severely compromised (DeLong et al., 2022). In the ischemic zone, dead and dying cells release molecules, which are known as Danger associated molecular patterns (DAMPs), into the surrounding environment. Microglia, which are the first immune cells to aggregate in this area (Long et al., 2023a), become activated and differentiate. Consequently, they release inflammatory factors that initiate and exacerbate the inflammatory response (Hu et al., 2014). This inflammation triggered after IS is a critical mechanism that contributes to secondary neuronal damage (Zhao et al., 2021). Neuroinflammation is a central component of stroke pathophysiology and a key factor in the development of therapeutic strategies (Minutoli et al., 2016). During acute IS-induced inflammation, interleukin-1 (IL-1) plays a pivotal role as a key regulatory molecule in cytokine release and cell death. In addition, circulating polymorphonuclear neutrophils (PMNs) increase significantly within hours of IS onset, and their levels are positively correlated with the severity of the stroke (Bonaventura et al., 2016). The pathological mechanisms underlying IS are highly complex, which involve multiple forms of cell death, including necrosis, apoptosis, and pyroptosis (Long et al., 2023b). Among these mechanisms, pyroptosis remains particularly unclear.

Pyroptosis is a recently identified form of inflammatory programmed cell death; it is characterized by distinct features such as cell membrane rupture, cell swelling and lysis, and the release of inflammatory mediators (Wang and Kanneganti, 2021). This process can be triggered by various pathological factors, including inflammatory cytokines, oxidative stress and abnormal cholesterol metabolism. Studies have shown that pyroptosis is involved in various systemic diseases, such as psychoneurological disorders (Xue et al., 2022), cardiovascular diseases (Jeyabal et al., 2016), autoimmune diseases (You et al., 2022), and ischemia-reperfusion injury (Zheng et al., 2022). Thus, it plays a critical role in the inflammatory response after IS (Sheng-Kai et al., 2021). According to distinct activation mechanisms, pyroptosis can be classified into three pathways: classical, nonclassical, and Caspase-3-mediated (Li L. et al., 2022). In the early stages of injury, microglia release reactive oxygen species (ROS), which can damage neurons, oligodendrocytes, astrocytes, and blood vessels (Colton, 2009; Lee et al., 2004; Li et al., 2021). Under oxidative stress, ROS accumulate in the body, which leads to a significant increase in NLRP3 inflammasome activity and the hyperactivation of Caspase-1; meanwhile, gasdermin D (GSDMD) is efficiently cleaved (Ran et al., 2021). Caspase-1 not only cleaves GSDMD into its N-terminal (GSDMD-N) and C-terminal (GSDMD-C) fragments but also processes the precursors of IL-18 and IL-1β into their active pro-inflammatory forms. The cleaved GSDMD-N serves as a key indicator of pyroptosis pathway activation; it binds to the cell membrane to form pores that facilitate the release of inflammatory factors and mediate the inflammatory response, which is the classical pyroptosis pathway (Wang et al., 2020). Studies by D. Yang-Wei et al. have shown that the expression levels of pyroptosis-related molecules, such as NLRP1, NLRP3, IL-1β, and IL-18, are significantly elevated in cortical neurons after IS (Fann et al., 2017). These findings suggest that pyroptosis may serve as a potential therapeutic target for the treatment of IS.

Currently, the primary treatments for IS include intravenous thrombolysis and endovascular thrombectomy, which can effectively reduce disability rates when rapid reperfusion is achieved. However, the high cost of these treatments limits their widespread application, especially in developing countries (Campbell et al., 2019). Therefore, identifying and developing new and effective therapeutic agents for the treatment of IS are urgently needed.

A growing body of research has highlighted the unique advantages of traditional Chinese medicine (TCM) in the intervention of IS. TCM is characterized by its safety and tolerability (Li X.-H. et al., 2022) and has demonstrated multiple neuroprotective and reparative functions, including maintaining blood-brain barrier (BBB) integrity, reducing cerebral edema, and promoting neurogenesis and synaptogenesis (Cao et al., 2019). Piperine (Pip), which is a bioactive alkaloid derived from black pepper (*Piper nigrum L.*) and long pepper (*Piper longum L.*), has attracted considerable attention due to its diverse biological effects, such as anti-tumor, anti-inflammatory, and anti-apoptotic properties (Smilkov et al., 2019). Notably, Pip not only enhances neuroprotection and repair in neurobehavioral deficit models induced by lipopolysaccharide (LPS) (Jangra et al., 2016) but also attenuates apoptosis by inhibiting Caspase-3 protein expression (Zheng, 2009). Furthermore, Pip has been shown to significantly improve motor coordination and cognitive function in animal models of Parkinson’s disease (PD), which effectively mitigates the inflammatory response by inhibiting dopaminergic neuron loss and reducing microglia activation (Qu and Yang, 2016). Despite the diversity of existing first-line therapeutic options for IS, critical clinical challenges remain, including the limited treatment time window and the risk of ischemia-reperfusion injury. Therefore, further in-depth studies on the potential neuroprotective mechanisms of Pip, particularly its regulation of the pyroptosis pathway to attenuate IS injury, may provide novel strategies for the clinical treatment of IS.

In this study, we employed the rat permanent middle cerebral artery occlusion (pMCAO) model and the glial BV-2 oxygen–glucose deprivation (OGD) model to investigate the protective mechanisms of Pip-mediated regulation of the Caspase-1 pyroptosis pathway against IS injury. Our findings aim to provide new theoretical and experimental foundations for the development of therapeutic strategies for IS.

## 2 Materials and methods

### 2.1 Drugs

Pip (Shanghai Desborne Biotechnology Co., Ltd., China, Catalog No. H441011), Nimodipine (MCE, United States, Catalog No. HY-B0265), and Caspase-1 inhibitor Ac-YVAD-cmk (MCE, United States, Catalog No. HY-16990) were used in this study. Pip was administered at a concentration of 30 mg/kg, while Nimo was administered at a concentration of 12 mg/kg; these concentrations are consistent with previously established protocols (Zhang Y. et al., 2022).

### 2.2 Animals and cells

In this study, specific pathogen-free healthy adult male Sprague–Dawley (SD) rats were selected for *in vivo* experiments. The rats weighed between 280 and 320 g and were aged 8–12 weeks. The animal production license number was SCXK [NNING]2020-0001. The experimental animals were housed in a barrier environment with a temperature of 23 ± 1°C and a relative humidity of 55% ± 5% while adhering strictly to a light/dark cycle for 12 h. The animals were provided with ample sterile drinking water and standard feed. The aforementioned experimental animals were supplied by the Laboratory Animal Center of Ningxia Medical University and were reviewed and approved by the center’s Animal Ethics and Welfare Committee (Ethics Code: IACUC-NYLAC-2021-G017).

For the *in vitro* experiments, the mouse microglial cell line-BV-2 cells (Zhejiang Meisen Cell Technology Co., Ltd., China, Catalog No. CTCC-003-0003)-was selected. The cells were cultured in Dulbecco’s modified Eagle medium (Gibco, United States) supplemented with 10% fetal bovine serum (FBS, Pernoside, China) and 1% penicillin-streptomycin mixture (Solepol, China), and maintained in a humidified incubator at 37°C with 5% CO_2_. Subculturing was performed when the cell density reached over 80% confluence.

### 2.3 Permanent MCAO model and OGD model

*In vivo* experiments utilized the pMCAO model to simulate IS. Rats were intraperitoneally injected with 2% sodium pentobarbital (0.2 mL/100 g) and secured in a supine position on a thermostatic rat dissection table at 37°C when they were anesthetized. The neck skin of the rats was fully exposed and disinfected with alcohol, followed by a midline incision along the neck to bluntly dissect the muscle and fascial tissues. The right common carotid artery (CCA), external carotid artery (ECA), and internal carotid artery (ICA) were exposed and isolated. The distal end of the ECA and the proximal end of the CCA were ligated with sutures. A small incision was made at the distal end of the CCA using scissors. A filament was inserted from the incision into the ICA to a depth of 17–18 mm. This procedure was followed by ligation of the CCA. The muscle and skin were sutured layer by layer, and penicillin (100,000 units per rat) was injected intraperitoneally. The rats were then placed on a thermostatic blanket at 37°C to recover, after which they were assessed using the Zea-Longa score. Rats with a score of 2 were selected as models for subsequent experiments. The Sham group underwent the same surgical procedure, but the middle cerebral artery was not occluded.

For *in vitro* experiments, OGD was used to simulate IS. BV-2 cells were cultured in a thermostatic incubator at 37°C until the cell density reached over 80%. The complete medium in the culture flask was then replaced with glucose-free medium. The flask was placed in a tri-gas incubator under conditions of 37°C, 97% N_2_, 5% CO_2_, and 2% O_2_ for 4 h to establish the OGD model of the cells.

### 2.4 Experimental design and drug administration

*In vivo* experiments: Excluding the Sham group, 54 SD rats with a Zea-Longa score of 2 were randomly divided into three groups: the Model (pMCAO) group, the Pip (pMCAO + Pip 30 mg/kg) group, and the Nimo (pMCAO + Nimo 12 mg/kg) group. After pMCAO, the Sham group and the Model group were administered 0.9% saline (0.01 mL/g), while the treatment groups received their respective drugs. All treatments were administered via oral gavage every 24 h for 14 consecutive days.

*In vitro* experiments: The cells were divided into the following groups: the control (Con) group, the OGD group, the OGD + Pip 50 μg/mL (Wang-Sheng et al., 2017) group, the OGD + Nimo 4 μg/mL (Li et al., 2019) group, the OGD + Ac-YVAD-cmk group, and the OGD + Ac-YVAD-cmk + Pip group.

### 2.5 Body weight measurement

The body weight of the rats was recorded daily for 14 days, and the changes in body weight were calculated relative to their weight after pMCAO. A body weight change graph was then plotted to visualize the trends over the experimental period.

### 2.6 Behavioral experiments

Behavioral tests were conducted on days 3, 6, 9, 12, and 14 after pMCAO. The assessments included the Zea-Longa neurological function score, the modified Neurological Severity Score (mNSS), the Postural Reflexes Score, and the Forelimb Grip Strength Test for rats in each group. The mNSS encompassed evaluations of motor function, sensory response, balance beam performance, reflex loss, and abnormal movements, with a maximum total score of 18 points. A lower score indicated a milder degree of neurological deficit in the rats.

### 2.7 Measurement of infarct area

On the 15th day after pMCAO, fresh brain tissues from the rats were extracted and sliced into 2 mm-thick sections. The slices were immersed in 1.5% 2,3,5-triphenyltetrazolium chloride (TTC) solution and incubated in a warm chamber at 37°C until staining was complete. The staining solution was then removed, and the brain slices were fixed in 4% paraformaldehyde (PFA) overnight. Photographs were captured to document the results. In the stained brain slices, the red areas represented normal brain tissue with adequate blood supply, while the white areas indicated ischemic brain tissue.

### 2.8 Histopathological observation

On the 15th day after pMCAO, the rats were euthanized and perfused, after which the brain tissues were acquired. The brain tissue sections were fixed, dehydrated, embedded in paraffin, and sectioned for hematoxylin–eosin (HE) staining. The stained sections were examined under a microscope (Olympus DP80, Japan) for histological analysis.

### 2.9 Transmission electron microscope

The brain tissues of rats were dissected to isolate the ischemic penumbra, preserved in fixative, and processed through fixation and dehydration before being embedded in epoxy resin. The embedded blocks were cut into ultrathin sections, which were then treated with uranyl acetate and lead citrate. Neuronal morphology was subsequently observed by transmission electron microscopy (TEM).

### 2.10 Western blot analysis

Tissues or cells from each group were homogenized in a grinding tube with grinding beads, and total protein was extracted using a Whole Protein Extraction Kit (Jiangsu KGI Biologicals, China). Protein concentration was determined by measuring absorbance using a fully automated enzyme immunoassay analyzer (Thermo Scientific, United States). Subsequently, 30 μg of protein was separated by sodium dodecyl sulfate–polyacrylamide gel electrophoresis (SDS-PAGE) and transferred onto polyvinylidene difluoride (PVDF) membranes. The membranes were blocked with 5% skimmed milk for 2 h at room temperature. Then, they were incubated overnight at 4°C with the following primary antibodies: Caspase-1 (1:1000; Santa Cruz, United States), cleaved Caspase-1 (1:1000; Absin, China), NLRP3 (1:1000; Shenyang Wanclass Science and Technology Co., Ltd., China), IL-1β (1:1000; Abcam, United Kingdom), and GSDMD-N (1:1000; Affinity, United States). After washing, the membranes were incubated with appropriate secondary antibodies for 1 h at room temperature. Protein bands were visualized using an enhanced chemiluminescence kit (Thermo Scientific, United States) and analyzed using ImageJ software.

### 2.11 Reverse transcription-polymerase chain reaction

Total RNA was extracted from tissues or cells using an RNA extraction kit (TIANGEN, China), which was followed by quantification of RNA concentration. Reverse transcription was performed using 2× SYBR Green I PCR Master Mix (ABclonal, China). Amplification was conducted using reverse transcription-polymerase chain reaction (RT-PCR) primers (Servicebio, China). Gapdh was used as an internal reference gene, and the Cycle threshold value of the target gene was compared with Gapdh. The relative mRNA expression levels of the target genes were calculated using the 2^−ΔΔCT^ method. The primer sequences for gene detection are listed in Table 1.

**TABLE 1:** Primer sequences for RT-PCR.

Primer name	Primer sequences (5′-3′)	Amplicon lengths (bp)
R-GSDMD-S	CAG​GCA​GCA​TCC​TTG​AGT​GTC	186
R-GSDMD-A	CCA​AGA​CGT​GCT​TCA​CCA​ACT	
R-NLRP3-S	GAT​TTC​TCC​ACA​ACT​CAC​CCA​A	112
R-NLRP3-A	AGT​CTG​GAA​GAA​CAG​GCA​ACA​T	
R-Il-1β-S	TGT​GAC​TCG​TGG​GAT​GAT​GAC	160
R-Il-1β-A	CCA​CTT​GTT​GGC​TTA​TGT​TCT​GTC	
R-IL-18-S	AAC​AGC​CAA​CGA​ATC​CCA​GAC	123
R-IL-18-A	TTG​TTT​TTA​CAG​GAG​AGG​GTA​GAC​A	
R-Caspase-1-S	TGT​AAT​GAA​GAC​TGC​TAC​CTG​GC	251
R-Caspase-1-A	CGA​GTG​GGT​GTT​TTC​ATT​ATT​GG	
R-ASC-S	ACT​ATC​TGG​AGG​GGT​ATG​GCT​T	189
R-ASC-A	CAA​TGA​GTG​CTT​GCC​TGT​GTT	
R-GAPDH-S	CTG​GAG​AAA​CCT​GCC​AAG​TAT​G	138
R-GAPDH-A	GGT​GGA​AGA​ATG​GGA​GTT​GCT	

### 2.12 Statistical analysis

All data was presented as Mean ± SEM. GraphPad Prism 9 software was utilized for statistical analysis. One-way ANOVA was applied for comparison between groups. *P* < 0.05 indicated statistical significance.

## 3 Results

### 3.1 Pip improves neurological function and motor performance in pMCAO rats

Neurological function and motor performance in rats were evaluated using the Zea-Longa neurological function score, mNSS, postural reflex score, beam walking test, and forelimb grip strength test. These assessments were conducted on days 3, 6, 9, 12, and 14 after pMCAO.

**FIGURE 1:**
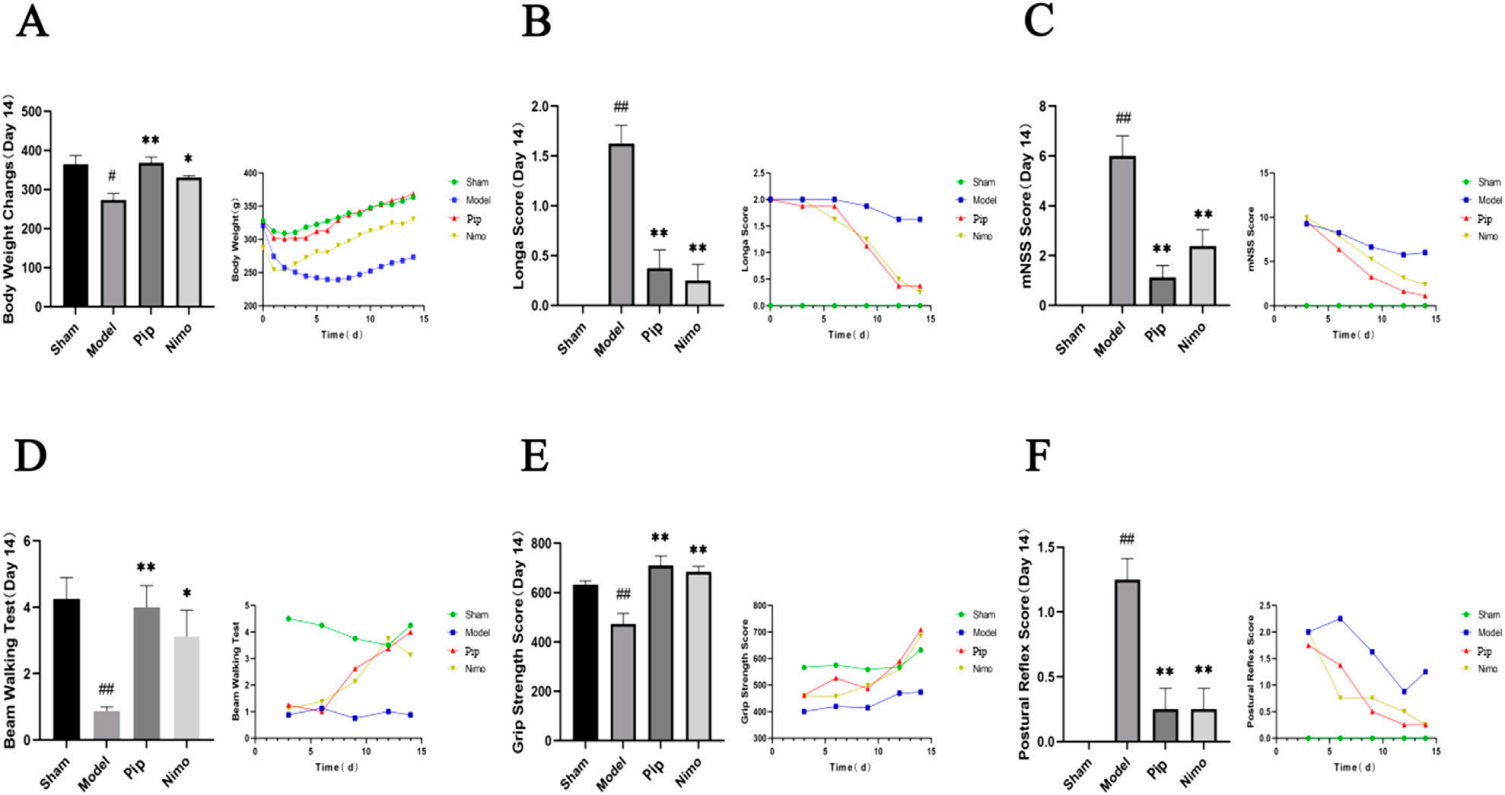
Effects of Pip on body weight, neurological function and motor performance in pMCAO rats. **(A)** Comparison of body weight among groups on day 14 post-model establishment and changes in body weight over time. **(B)** Comparison of Zea-Longa scores among groups on day 14 post-model establishment and changes in Zea-Longa scores over time. **(C)** Comparison of mNSS scores among groups on day 14 post-model establishment and changes in mNSS scores over time. **(D)** Comparison of Beam Waliking Test scores among groups on day 14 post-model establishment and changes in Beam Waliking Test scores over time. **(E)** Comparison of forelimb grip strength among groups on day 14 post-model establishment and changes in forelimb grip strength over time. **(F)** Comparison of posture reflex scores among groups on day 14 post-model establishment and changes in posture reflex scores over time. Compared to the Sham group, ^*#*^ *P* < 0.05, ^*##*^ *P* < 0.01; compared to the Model group, *P < 0.05, **P < 0.01 (*n* = 8 per group).

As shown in Figure 1A, the Sham group exhibited an overall increasing trend in body weight, with a transient decrease in the earlier stage likely due to surgical trauma. By contrast, the Model group showed a significant decrease in body weight compared with the Sham group (*P* < 0.05). However, the body weight of the Pip group increased significantly compared with that of the Model group (P < 0.01), and a similar trend was observed in the Nimo group (P < 0.05). These findings suggest that IS induced weight loss in rats, which was mitigated by Pip treatment.

Based on the Zea-Longa score (Figure 1B), mNSS score (Figure 1C), and postural reflex score (Figure 1F), the scores of rats in the Model, Pip, and Nimo groups were comparable in the early postoperative period. However, significant differences emerged from day 6 onward, particularly on day 14. The Model group exhibited significantly higher scores than the Sham group (P < 0.01), while the Pip and Nimo groups showed significantly lower scores than the Model group (P < 0.01). In the beam walking test (Figure 1D), the Pip and Nimo groups performed similarly to the Model group before day 6. However, after day 6, the scores of the Pip and Nimo groups increased significantly (P < 0.05). In addition, the forelimb grasping tension (Figure 1E) in the Pip and Nimo groups increased significantly (P < 0.01). Although the Model group also showed some improvements in forelimb grip strength test, it remained significantly lower than the Sham group (*P* < 0.01), which is likely due to the limited self-healing capacity of rats in this group. These results indicate that IS leads to impaired motor ability and strength in rats. However, Pip intervention significantly improved neurological dysfunction and motor deficits, which suggests that Pip has a protective effect against cerebral ischemic neurological injury in rats. These data indicate that Pip has a protective effect against neural damage caused by cerebral ischemia in rats.

### 3.2 Pip reduces cerebral infarction volume in pMCAO rats

**FIGURE 2:**
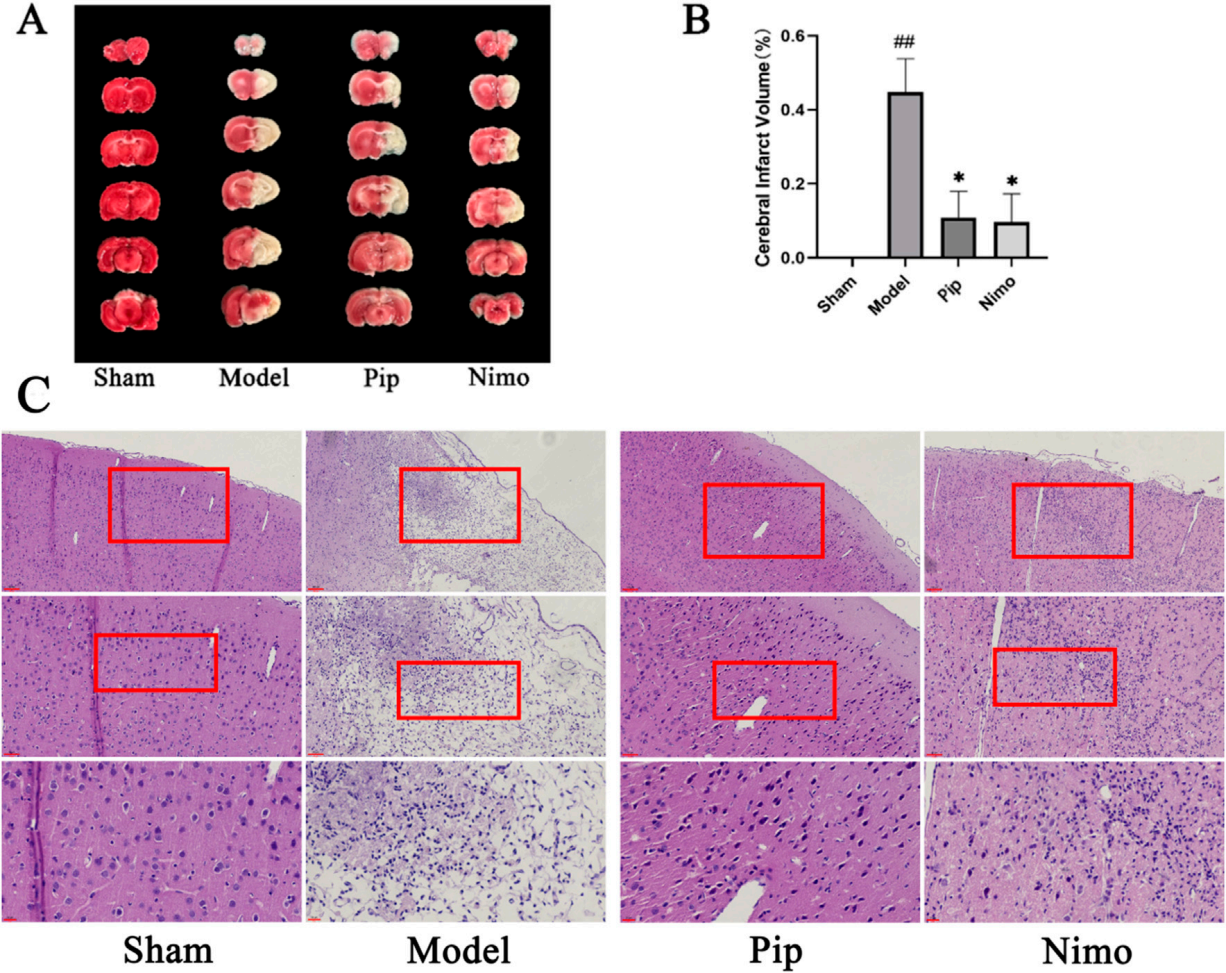
Effects of Pip on cerebral infarction volume and pathological damage in pMCAO rats. **(A)** Photographs of TTC-stained brain tissue sections. **(B)** Statistical analysis of cerebral infarction volume in each group. Compared to the Sham group, ^*#*^ *P* < 0.05, ^*##*^ *P* < 0.01; compared to the Model group, *P < 0.05, **P < 0.01 (*n* = 3 per group). **(C)** HE staining. Scale bar = 20 μm at ×400 magnification; scale bar = 50 μm at ×200 magnification; scale bar = 100 μm at ×100 magnification. The scale bars are indicated at the bottom left corner of the images (*n* = 3 per group).

TTC staining is a commonly used method to assess cerebral ischemic injury by measuring the volume of cerebral infarction. On the 15th day after pMCAO, brain tissue samples were collected from each group of rats and subjected to TTC staining (Figure 2A). As shown in Figure 2B, the cerebral infarction volume in the Sham group was 0; compared with the infarction area in the Sham group, that in the Model group significantly increased (P < 0.01); compared with that in the Model group, the infarction areas in the Pip and Nimo groups showed varying degrees of reduction (P < 0.05). These results indicate that Pip intervention can reduce the volume of cerebral infarction.

### 3.3 Pip improves cerebral pathological morphology in pMCAO rats

The results of HE staining are shown in Figure 2C. In the Sham group, the cytoplasm of brain tissue cells was uniformly stained, with cells arranged neatly and evenly distributed in the cytoplasm. Compared with the Sham group, the cytoplasm staining in the Model group became lighter, the number of cells decreased, and significant nuclear pyknosis was observed. In contrast to the Model group, the Pip and Nimo groups exhibited reduced cellular edema, uniform cytoplasmic staining, an increased number of cells, and a noticeable recovery in cell morphology. These results demonstrate that Pip can alleviate cerebral ischemic injury.

### 3.4 Pip alleviates neuronal damage in pMCAO rats

**FIGURE 3:**
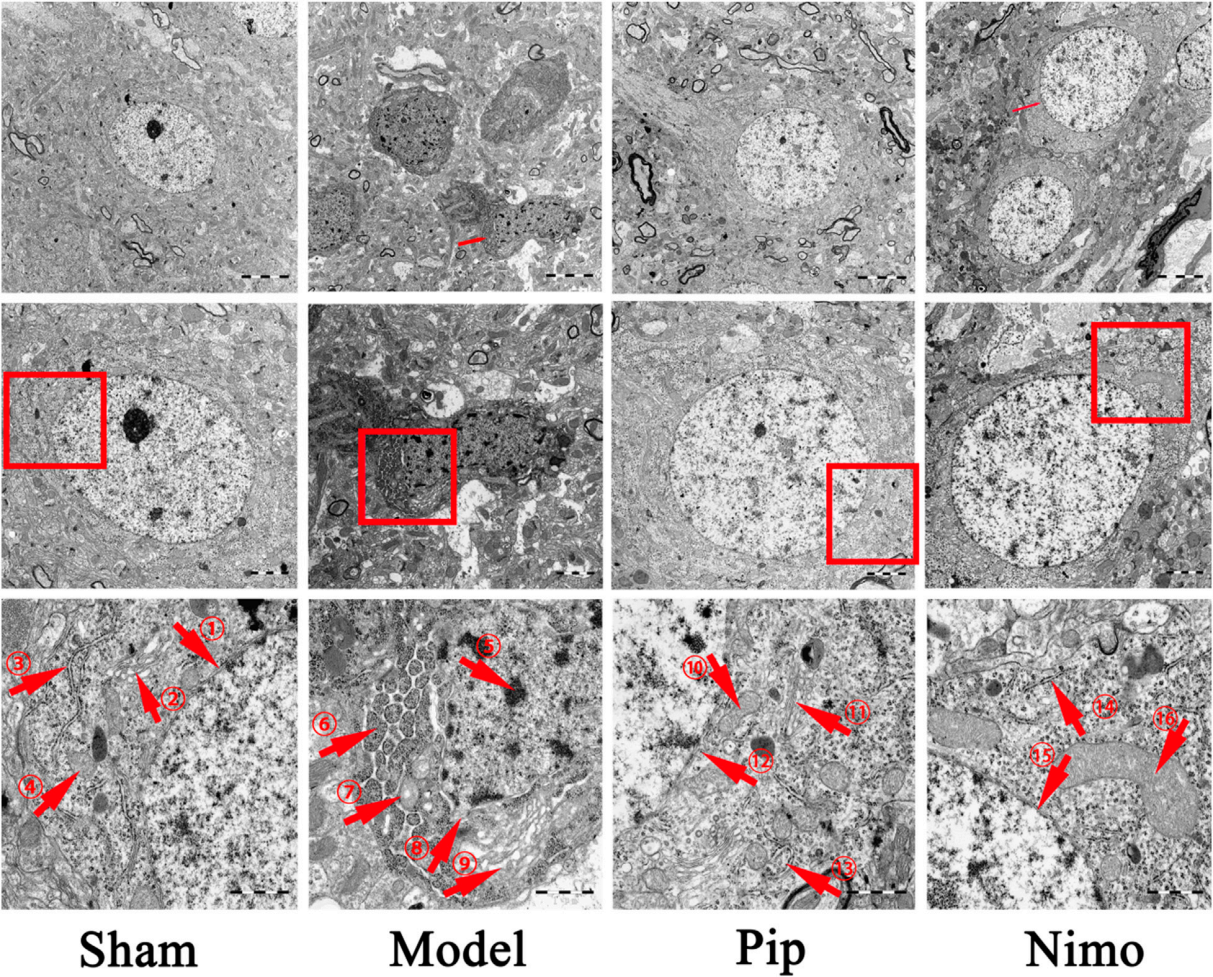
Effects of Pip on neuronal cell damage in pMCAO rats. Ultrastructure of neurons in the ischemic penumbra. ①⑫⑮ represent the double-layered nuclear membrane structure; ②⑪ represent Golgi apparatus; ③⑬⑭ represent rough endoplasmic reticulum; ④⑩⑯ represent mitochondria; ⑤-⑨ respectively represent heterochromatin, swollen and structurally damaged mitochondria, fragmented nuclear membrane structures, and swollen Golgi apparatus. Scale bar = 5 μm at 5000× magnification; scale bar = 2 μm at ×10000 magnification; scale bar = 1 μm at ×30000 magnification. The scale bars are indicated at the bottom right corner of the images (*n* = 3 per group).

The TEM results are shown in Figure 3. In the Sham group, the double-membrane structure of the neuronal nuclei was clear, with regular and intact boundaries, and the distribution and structure of organelles in the cytoplasm were normal. In the Model group, the neuronal nuclei exhibited pyknosis, incomplete nuclear membranes, and heterochromatin deposition beneath the nuclear membrane. Moreover, the endoplasmic reticulum was swollen, with ribosomes attached to it detaching. The Golgi apparatus was also dilated. Furthermore, the double-membrane structure of the mitochondria was blurred, with swollen cristae and matrix. Autophagic lysosomes were visible in the cytoplasm, and dendritic edema with loss of contents was observed. Compared with the Model group, the Pip and Nimo interventions significantly improved the extent of neuronal damage. This improvement was evidenced by intact nuclear membranes with clear boundaries, as well as well-defined structures with reduced swelling of mitochondria, endoplasmic reticulum, and other organelles.

### 3.5 Pip reduces protein and mRNA expression of pyroptosis-related factors

**FIGURE 4:**
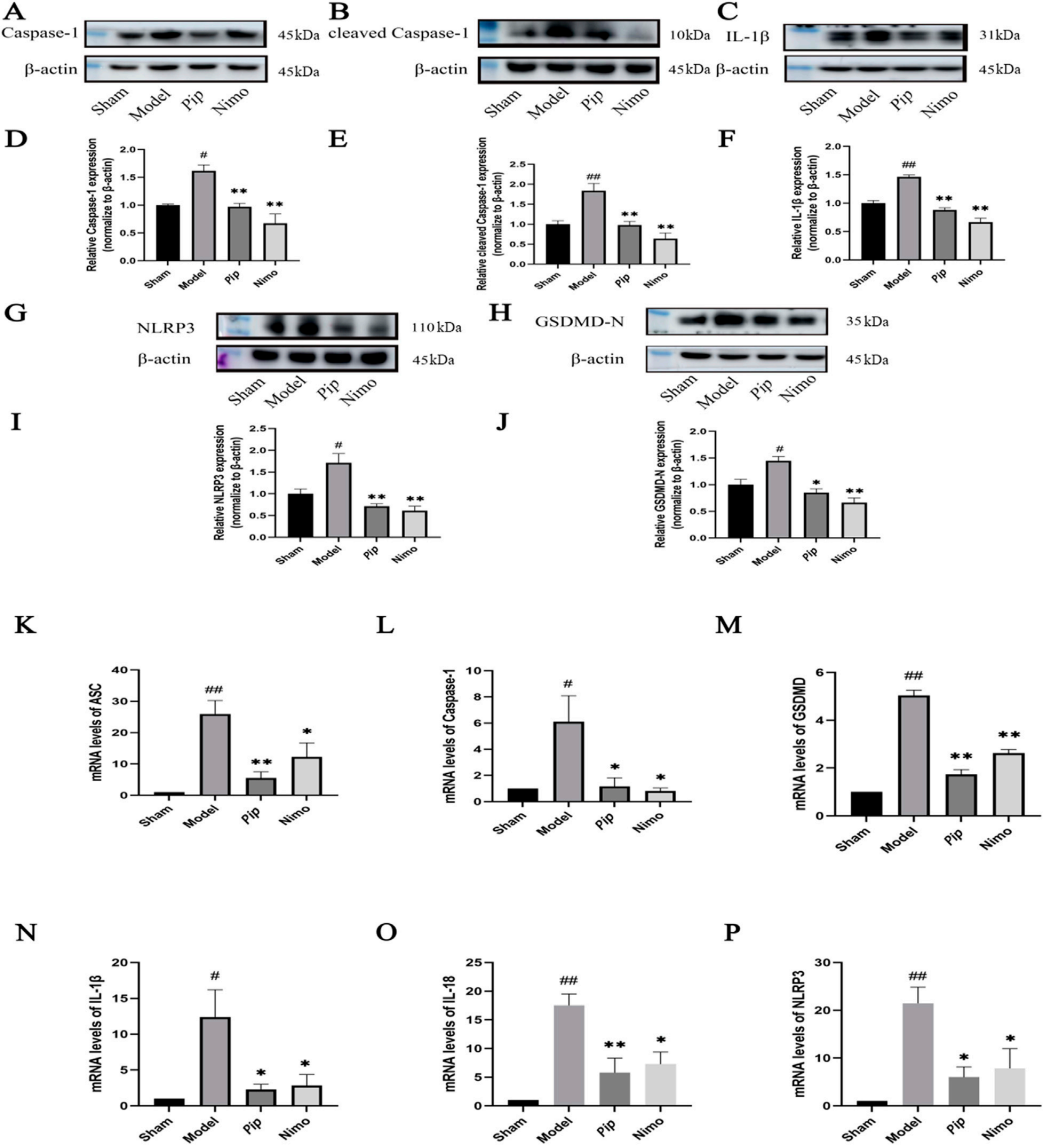
Effects of Pip on the protein and mRNA expression of pyroptosis-related factors in ischemic brain tissue. **(A–C,G,H)** Western blot bands of Caspase-1, cleaved Caspase-1, IL-1β, NLRP3 and GSDMD-N in each group. **(D–F,I,J)** Bar graphs showing the target protein/β-actin ratios in each group. **(K–P)** Bar graphs showing the mRNA expression levels of ASC, Caspase-1, GSDMD, IL-1β, IL-18 and NLRP3 in each group. Compared to the Sham group, ^*#*^ *P* < 0.05, ^*##*^ *P* < 0.01; compared to the Model group, *P < 0.05, **P < 0.01 (*n* = 4 per group).

Pyroptosis-related factors play a crucial role in cellular pyroptosis. As shown in Figures 4A–J, in the pMCAO model, the protein expression levels of Caspase-1, NLRP3, GSDMD-N (P < 0.05), cleaved Caspase-1, and IL-1β (P < 0.01) were significantly increased in the Model group compared with those in the Sham group. Compared with the Model group, the Pip group exhibited significantly reduced protein expression levels of Caspase-1, cleaved Caspase-1, NLRP3, IL-1β (P < 0.01), and GSDMD-N (P < 0.05). Meanwhile, the Nimo group also showed decreased protein expression levels of Caspase-1, NLRP3, GSDMD-N, cleaved Caspase-1, and IL-1β (P < 0.01). Similarly, RT-PCR results (Figures 4K–P) indicated that the mRNA expression levels of ASC, GSDMD, IL-18, NLRP3 (P < 0.01), Caspase-1, and IL-1β (P < 0.05) were significantly elevated in the Model group compared with those in the Sham group. Compared with the Model group, the Pip and Nimo groups exhibited significant downregulation in the mRNA expression levels of ASC, GSDMD, IL-18, NLRP3, Caspase-1, and IL-1β (P < 0.05).

**FIGURE 5:**
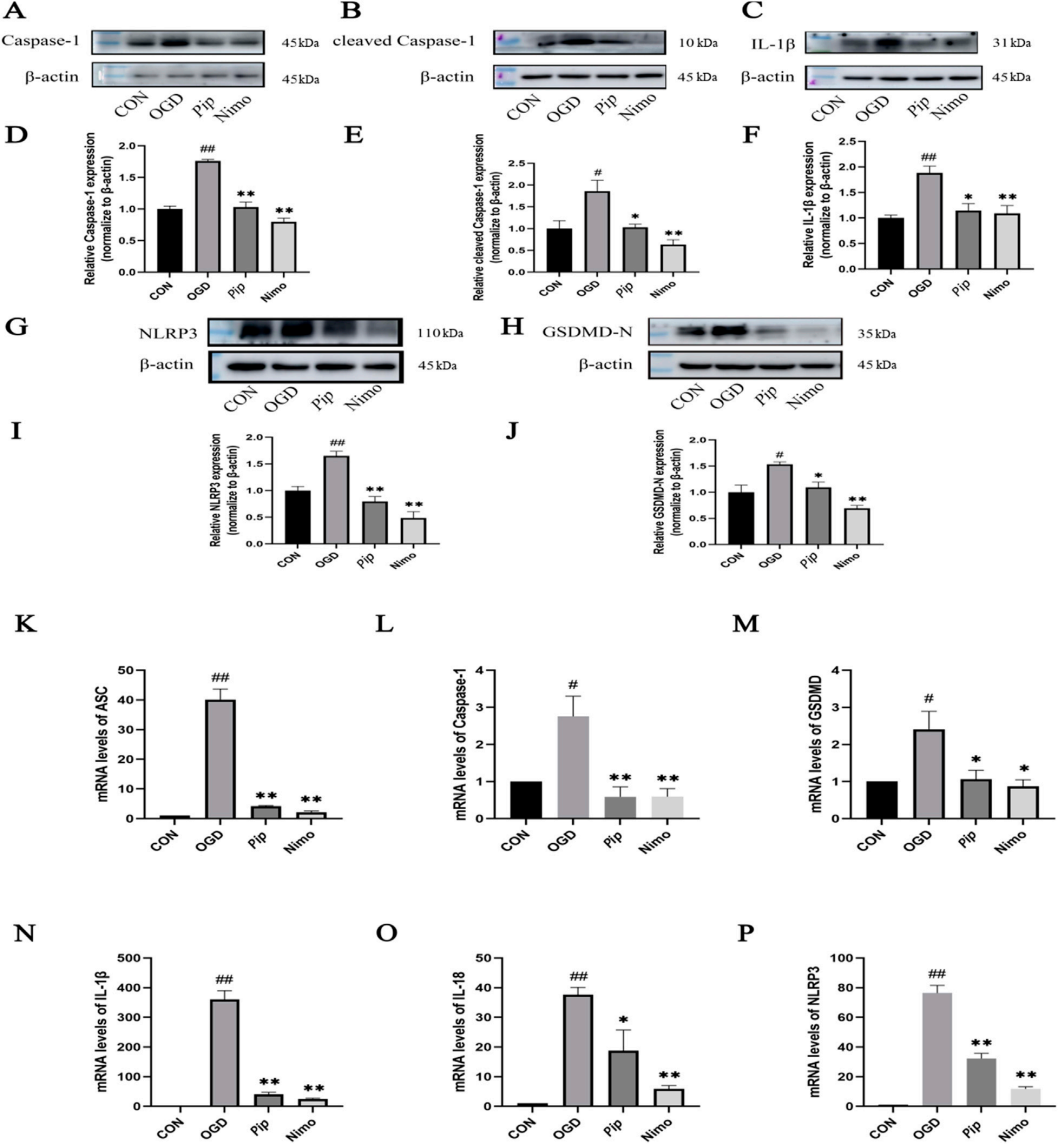
Effects of Pip on the protein and mRNA expression of pyroptosis-related factors in BV-2 cells. **(A–C,G,H)** Western blot bands of Caspase-1, cleaved Caspase-1, IL-1β, NLRP3 and GSDMD-N in each group. **(D–F,I,J)** Bar graphs showing the target protein/β-actin ratios in each group. **(K–P)** Bar graphs showing the mRNA expression levels of ASC, Caspase-1, GSDMD, IL-1β, IL-18 and NLRP3 in each group. Compared to the Con group, ^*#*^ *P* < 0.05, ^*##*^ *P* < 0.01; compared to the OGD group, *P < 0.05, **P < 0.01 (*n* = 4 per group).

BV-2 cells were treated with OGD induction, followed by Western blot (Figures 5A–J) and RT-PCR (Figures 5K–P), to further validate the aforementioned findings. Consistent with the *in vivo* results, the protein and mRNA expression levels of factors such as Caspase-1 and GSDMD-N were significantly increased in the OGD group (P < 0.05) compared with those in the Con group. Compared with the OGD group, the Pip and Nimo groups showed significantly reduced protein and mRNA expression levels of Caspase-1, GSDMD-N, and other factors (P < 0.05).

### 3.6 Pip alleviates pyroptosis by mediating Caspase-1

**FIGURE 6:**
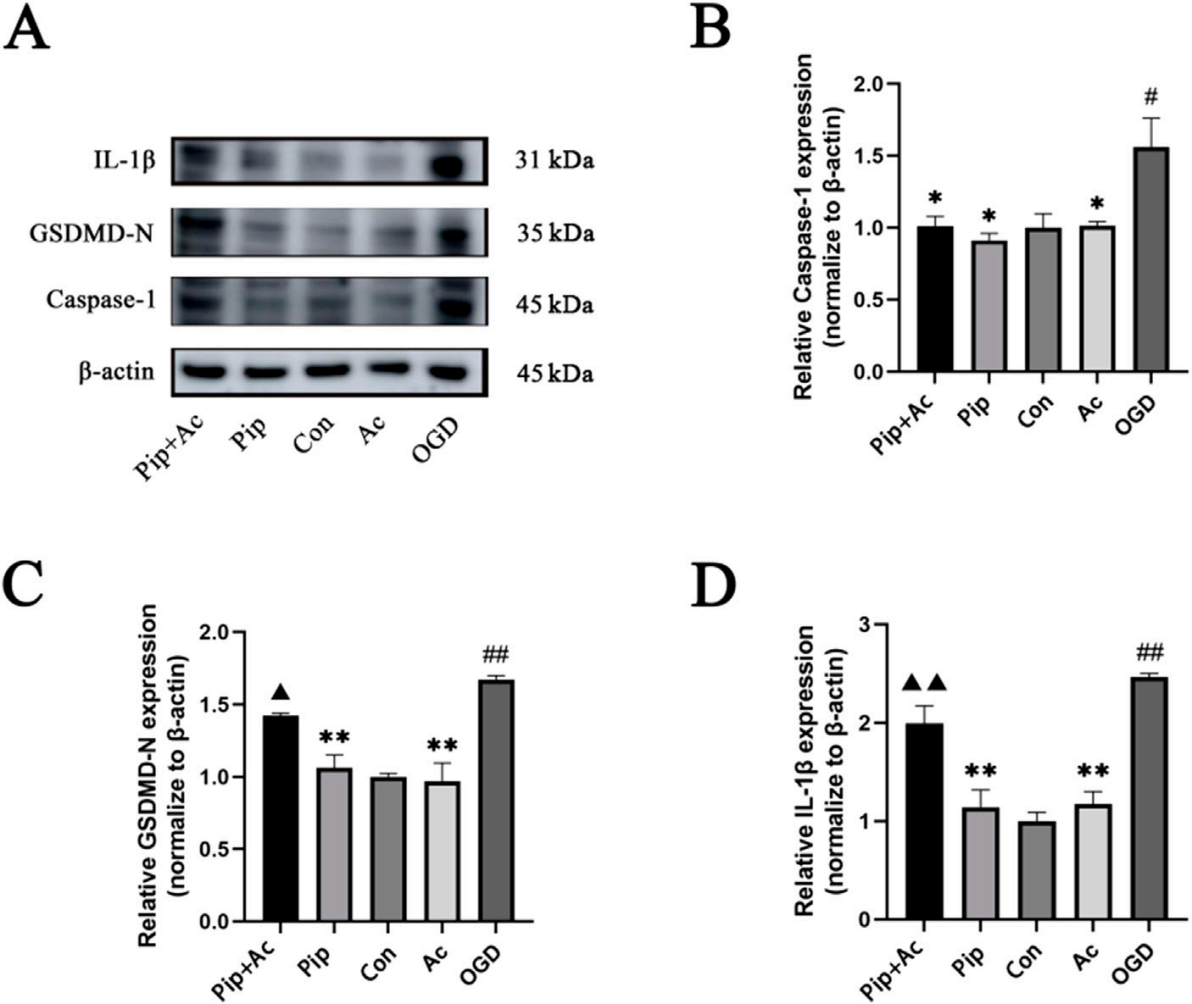
Effects of Ac-YVAD-cmk on Pip’s inhibition of pyroptosis. **(A)** Western blot bands and bar graphs of Caspase-1, GSDMD-N and IL-1β in the Pip + Ac-YVAD-cmk group, Pip group, Con group, Ac-YVAD-cmk group and OGD group. **(B–D)** Bar graphs showing the protein expression levels of Caspase-1, GSDMD-N and IL-1β in the Pip + Ac-YVAD-cmk group, Pip group, Con group, Ac-YVAD-cmk group and OGD group. Compared to the Con group, ^*#*^ *P* < 0.05, ^*##*^ *P* < 0.01; compared to the OGD group, *P < 0.05, **P < 0.01; compared to the Pip group, ^*▲*^ *P* < 0.05, ^*▲▲*^ *P* < 0.01 (*n* = 4 per group).

Caspase-1 can cleave GSDMD into GSDMD-N and mature IL-1β, with GSDMD-N serving as an indicator protein for pyroptosis pathway activation. Therefore, we further investigated whether Pip could inhibit pyroptosis by regulating Caspase-1. Using the Caspase-1 inhibitor Ac-YVAD-cmk in the BV-2 cell OGD model, we validated the protein expression levels of Caspase-1, GSDMD-N, and IL-1β through Western blot (Figure 6). We found that the protein expression level of Caspase-1 was significantly decreased in the Pip + Ac-YVAD-cmk group (P < 0.05) compared with that in the OGD group. Compared with the Pip group, the combined intervention of Pip and Ac-YVAD-cmk significantly increased the protein expression levels of GSDMD-N (P < 0.01) and IL-1β (P < 0.05). Meanwhile, the protein expression level of Caspase-1 showed no significant difference (P > 0.05). This result indicates that inhibiting Caspase-1 attenuated the suppressive effect of Pip on the protein expression of downstream factors GSDMD-N and IL-1β. Compared with the Con group, the OGD group exhibited increased protein expression levels of Caspase-1, GSDMD-N, and IL-1β. Compared with the OGD group, the Pip group and Ac-YVAD-cmk group showed decreased protein expression levels of Caspase-1, GSDMD-N, and IL-1β. These results confirm that Pip alleviates pyroptosis in BV-2 cells post-OGD by regulating Caspase-1 to inhibit the activation of GSDMD and the maturation of IL-1β.

## 4 Discussion

Previous studies have demonstrated that the dichloromethane fraction (DF) of *Piper nigrum L.* and *P. longum L.* can reduce brain injury and suppress apoptosis and autophagy by activating the Akt-mTOR signaling pathway (Zhang et al., 2020) and inhibiting the expression of autophagy-related proteins ATG5-ATG12 family, BECLIN1-VPS34 family, and LC3 (Yuan et al., 2022) in IS. Furthermore, tetrahydropiperine (THP) and Pip can also alleviated neurological damage in IS by activating the PI3K/Akt/mTOR pathway and inhibiting autophagy (Ren et al., 2024; Zhang Y. et al., 2022). These findings collectively confirm that piper extracts exhibit significant neuroprotective effects in both *in vivo* and *in vitro* models of IS. To further elucidate Pip’s mechanisms of action, this study evaluated the impact of Pip on the pyroptosis pathway involved in cerebral ischemia through *in vivo* and *in vitro* experiments. Behavioral and morphological results demonstrated that Pip significantly improves neurological function, reduces cerebral infarction volume, and alleviates neuronal damage. Western blot and RT-PCR results further confirmed that Pip effectively lowers levels of inflammatory response and oxidative stress, inhibits Caspase-1 expression, and subsequently attenuates the inhibitory effects of Pip on downstream factors of Caspase-1. These data suggest that Pip may exert its neuroprotective effects by inhibiting the Caspase-1-mediated pyroptosis pathway.

Pip has demonstrated significant anti-inflammatory and antioxidant potential in various neurological disorders. Studies have confirmed that Pip significantly improves motor coordination and enhances learning and memory capabilities in a PD mouse model by inhibiting neuroinflammatory responses. It also effectively reduces the degeneration and loss of dopaminergic neurons while markedly decreasing the activation of microglia and the expression levels of inflammatory factors (Qu and Yang, 2016). In Alzheimer’s disease (AD) research, Pip alleviates cognitive impairment by combating oxidative stress (Nazifi et al., 2020). In addition, in a mouse model of spinal cord injury (SCI), Pip promotes functional recovery post-SCI by inhibiting autophagy-mediated inflammation and pyroptosis (Zhang H. et al., 2022). Pip also exerts neuroprotective effects by regulating the PI3K/AKT/mTOR pathway to inhibit autophagy (Zhang Y. et al., 2022). In this study, within the pMCAO model, Pip significantly improved neurological function and motor abilities. This improvement was evidenced by reduced posture reflex scores, improved balance beam scores, and increased forelimb grip strength. Furthermore, Pip intervention led to a significant increase in rat body weight, which indicates that Pip effectively enhances the overall physiological condition of IS rats.

In IS, BBB damage plays a crucial role in the secondary deterioration of neurological function, with its disruption closely linked to ischemia-induced inflammation and cerebral edema formation (Bellut et al., 2021). Research has shown that nanoparticle intervention significantly alleviates pyroptosis and inflammatory responses, which reduces tissue edema after traumatic brain injury (Zhang et al., 2024). Pooja Kaushik and colleagues also found that, in a transient middle cerebral artery occlusion model (tMCAO), Pip not only inhibits mitochondrial dysfunction but also reduces the expression of pro-inflammatory factors in the cerebral cortex, which suppresses neuronal apoptosis (Kaushik et al., 2021). The experimental results of the present study confirm that, compared with the Model group, Pip treatment significantly reduces cerebral infarction volume, alleviates tissue edema, increases cell count, and markedly restores cell morphology. Neuronal nuclear membranes remain intact, and cell boundaries are clear. Moreover, the structures of mitochondria, endoplasmic reticulum, and other organelles are well-defined, with reduced swelling.

Pyroptosis, as a pro-inflammatory programmed cell death mode, triggers a robust immune response through the release of inflammatory factors (Deng et al., 2024). The activation of inflammasomes and pyroptosis exacerbates ischemic injury, while the inhibition of inflammasome activation exhibits significant neuroprotective effects (Luo et al., 2022). Microglia, as the first immune cells to aggregate in the ischemic penumbra, release inflammatory molecules upon activation and intensify the inflammatory response. Our findings confirm that the protein and mRNA expression of IL-1β is significantly upregulated after pMCAO/OGD. Proteins containing Nucleotide-binding oligomerization domains (NODs) are collectively referred to as NOD-like receptors (NLR), with those having an N-terminal pyrin domain (PYD) known as the NLRP family (Amoushahi et al., 2019). These receptors are typical initiators of pyroptosis (Aizawa et al., 2020). The Caspase recruitment domain (CARD) binds to the CARD domain on pro-Caspase-1 to form the NLRP3 inflammasome (Wu et al., 2020). The NLRP3 inflammasome induces the self-cleavage of Caspase-1, with two dimers recruiting to form a tetramer. This tetramer cleaves GSDMD, and protease-mediated cleavage in the linker loop releases the N-terminal domain, which oligomerizes on the plasma membrane to form non-selective pores; this formation leads to changes in membrane permeability and cell swelling (Zhao et al., 2022). Additionally, Xin Guo and colleagues found that Pip can alleviate myocardial ischemia-reperfusion injury (MIRI) and pyroptosis in rats by modulating the miR-383/RP105/AKT pathway (Guo et al., 2020).

Moreover, Pip plays an important role in mitigating inflammatory responses. In a rat model of non-compressive lumbar disc herniation, the expression of miR-520a is positively correlated with anti-inflammatory cytokines, while the expression of P65 is positively correlated with pro-inflammatory cytokines. Moreover, miR-520a can specifically target P65. Yu Jiuwang and colleagues found that Pip promotes the expression of miR-520a; this promotion inhibits the expression of p65, which downregulates pro-inflammatory factors while upregulating anti-inflammatory factors; ultimately, pain is alleviated (Yu et al., 2021). Lu Hui and colleagues discovered that Pip reduces the expression of inflammatory factors and improves skin lesions caused by psoriasis (Lu et al., 2023). Obesity-induced insulin resistance (IR) and diabetes mellitus (DM) lead to metabolic-related inflammation; Pip can act as an immunomodulator by inhibiting the polarization of M1 macrophages in adipose tissue, which exerts anti-inflammatory effects to treat related diseases (Liu et al., 2020). In another experiment, Pip successfully inhibited the production of PGE2 and NO in IL-1β-induced human osteoarthritic chondrocytes (Ying et al., 2013). Pip can ameliorate hepatic steatosis and hepatocyte injury in mice with steatohepatitis while also reducing pyroptosis markers such as NLRP3, ASC, and Caspase-1 (Ran et al., 2024). In the current study, *in vivo* experimental results also confirmed that, compared with the Sham group, the Model group exhibited increased protein expression of Caspase-1, cleaved Caspase-1, NLRP3, IL-1β, and GSDMD-N. Compared with the Model group, the Pip group showed decreased protein expression of Caspase-1, cleaved Caspase-1, NLRP3, IL-1β, and GSDMD-N. Pengfei Xu and colleagues found that, in mice experiencing pyroptosis due to subarachnoid hemorrhage, treatment with 5Z-7-oxozeaenol (OZ) resulted in decreased mRNA levels of NLRP3, IL-1β, and IL-18 compared with those in untreated mice (Xu et al., 2021). Similarly, our experimental results indicate that Pip-treated pMCAO rats exhibited downregulated mRNA expression of NLRP3, IL-1β, and IL-18.

Yangshuo Tang and colleagues induced pyroptosis in HUVECs by using LPS-ATP *in vitro* (Tang et al., 2019). The mRNA levels of NLRP3, Caspase-1, ASC, GSDMD, IL-1β, and IL-18 were significantly higher in the LPS-ATP group than in the control group. However, the mRNA expression of these related factors was markedly suppressed in the lotusine-treated group. Furthermore, the changes in protein levels of NLRP3, cleaved Caspase-1, GSDMD-N, IL-1β, and IL-18 were consistent with the changes in mRNA levels. We employed BV-2 cells subjected to OGD to simulate *in vivo* ischemic injury for further validating this conclusion. The results of Western blot and RT-PCR experiments were consistent with the findings of Yangshuo Tang and colleagues. The results showed that the protein and mRNA expression of Caspase-1, IL-1β, and NLRP3 increased in the OGD group. Meanwhile, the protein and mRNA expression of NLRP3, Caspase-1, GSDMD, and IL-1β significantly decreased in the Pip group.

Dawei Dong and colleagues found that the expression of pyroptosis-related molecules Caspase-1, NLRP3, GSDMD, ASC, IL-1β, and IL-18 was upregulated in rats after distal middle cerebral artery occlusion (dMCAO). However, these molecules were downregulated in dMCAO rats treated with the Caspase-1 inhibitor VX-765. This result indicates that blocking Caspase-1 can inhibit pyroptosis and downregulate the expression of pyroptosis-related molecules (Dong et al., 2022). Similarly, we employed the Caspase-1 inhibitor Ac-YVAD-cmk to further validate whether Pip exerts its effects via Caspase-1-dependent mechanisms. The results demonstrated that inhibition of Caspase-1 significantly attenuated Pip’s suppressive effects on both GSDMD-N and IL-1β. This evidence confirms that Pip inhibits the activation of GSDMD-N and IL-1β through Caspase-1-mediated pathways, thereby substantiating our hypothesis.

In summary, the results of this study indicate that Pip may exert neuroprotective effects against IS injury by regulating the Caspase-1-mediated pyroptosis pathway. Focusing on pyroptosis provides new insights into the therapeutic potential of Pip for IS and offers a solid theoretical foundation for improving patient prognosis.

## 5 Conclusion

This study provides robust evidence for the protective effects of Pip against cerebral ischemic injury. Our findings demonstrate that Pip significantly ameliorates behavioral impairments, reduces neuronal damage, and suppresses inflammation by modulating the Caspase-1-mediated pyroptosis pathway. In the OGD model of BV-2 cells, Pip decreases the expression of pyroptosis-related factors such as GSDMD-N, which inhibits pyroptosis and mitigates cell injury. These discoveries not only enhance our understanding of the potential therapeutic effects of Pip but also open new avenues for its clinical application in the treatment of IS.

## Data Availability

The original contributions presented in the study are included in the article/Supplementary Material, further inquiries can be directed to the corresponding authors.

## Abbreviation

IS, ischemic stroke; pMCAO, permanent middle cerebral artery occlusion; CCA, common carotid artery; ECA, external carotid artery; ICA, internal carotid artery; Pip, piperine; Nimo, nimodipine; TEM, transmission electron microscopy; TTC, 2,3,5-triphenyltetrazolium chloride; HE, hematoxylin-eosin staining; CON, control; OGD, oxygen–glucose deprivation; SPF, specific pathogen-free; DMEM, dulbecco’s modified eagle’s medium; FBS, fetal bovine serum.
